# Comparison of anesthesia induction with ciprofol, propofol, and etomidate in elderly patients undergoing gastrointestinal endoscopy: a single center, prospective, double blind randomized controlled trial

**DOI:** 10.3389/fragi.2026.1842800

**Published:** 2026-08-03

**Authors:** Yingchao Guan, Gang Zhou, Conghui Wang, Yusong Lin, Minghong Ju, Wen He, Xiaodong Wang

**Affiliations:** 1 Department of Anesthesiology, Weihai Municipal Hospital, Cheeloo College of Medicine, Shandong University, Weihai, Shandong, China; 2 The Second School of Clinical Medicine of Binzhou Medical University, Yantai, Shandong, China

**Keywords:** ciprofol, etomidate, gastrointestinal endoscopy, propofol, randomized controlled trial

## Abstract

**Background:**

We compared benefits and adverse effects of anesthesia induction with ciprofol, propofol, and etomidate in elderly patients undergoing gastrointestinal endoscopy anesthesia.

**Methods:**

A total of 391 patients who underwent painless gastrointestinal endoscopy; age ≥ 60 years; ASA I-III were involved between April 30 and 30 December 2025. The primary outcome was hypoxemia or apnea during anesthesia. Secondary outcomes included systolic blood pressure (SBP), diastolic blood pressure (DBP), mean blood pressure (MBP), heart rate (HR), respiratory rate (RR), SpO_2_ during surgery, and adverse reactions such as coughing dizziness and agitation.

**Results:**

No differences were found in hypoxemia and apnea. Except for RR, other hemodynamic and respiratory parameters were significantly different among the groups (p < 0.05), and the degree of blood pressure decline in group E was smaller than groups C and P. Group E patients had lower intraoperative hypotension (p = 0.007) and lower demand for vasoactive drugs (p = 0.006). The injection pain scores in groups C and E were lower, but patients in group E had a higher incidence of myoclonus, and the proportion of coughing and hiccups during endoscopy was higher in group E. The incidence of dizziness was lower in Group C.

**Conclusion:**

No significant differences were observed in the incidence of hypoxemia and apnea among three groups. Etomidate has the advantage of stable hemodynamics; however, the incidence of myoclonus, bucking, and hiccups is high. Injection pain occurred less frequently with ciprofol and etomidate, while ciprofol was associated with a low incidence of postoperative dizziness.

**Clinical Trail Registration:**

https://www.chictr.org.cn/showproj.html?proj=269837, identifier ChiCTR2500101280.

## Introduction

The number of elderly patients undergoing surgery under anesthesia is increasing annually. In China, as of the end of 2024, the population aged 60 and above nationwide reached 310.31 million, accounting for 22.0% of the total population, while elderly population aged 65 and above was 220.23 millions, accounting for 15.6% of the total population (Ministry of Civil Affairs of the Peopl e’s Republic of China, 2024). Age is an independent risk factor for multiple perioperative complications, such as stroke, major adverse cardiac events, acute kidney injury, respiratory failure, and infections (Shu et al., 2025; Gong et al., 2023; Becerra-Bolaños et al., 2024); therefore, the increasing number of elderly patients poses new challenges to optimizing perioperative management. With the advancement of anesthesia technology and the emphasis on comfort, gastrointestinal endoscopy under sedation has gradually become the first choice for clinicians and patients; however, research indicates that elderly patients are independent risk factors for postoperative cardiovascular and cerebrovascular events and have an increased risk of lower respiratory infections and pneumonia, although these adverse reactions are usually mild (Martínez-González et al., 2025; Kim et al., 2020; Kollmann et al., 2018). Therefore, caution should be exercised when performing gastrointestinal endoscopy under sedation in elderly patients, and guidelines also suggest careful consideration when selecting sedatives (Sidhu et al., 2024).

Propofol is still widely used and is safe in the elderly population; however, it is associated with adverse reactions, such as hemodynamic fluctuations, respiratory depression, and injection pain (Lu et al., 2022; Ogawa et al., 2020; Ahmer et al., 2024). Etomidate has a relatively small impact on hemodynamics and a milder respiratory inhibitory effect than propofol; however, the incidence of myoclonus is significantly increased, which may affect the perioperative experience of examining physicians and patients (Liu et al., 2023). Ciprofol has gradually gained attention as a recently launched sedative drug because of its excellent sedative effects and fewer adverse reactions. A meta-analysis has found that ciprofol has hemodynamic stability, mild respiratory depression, and comparable efficacy to propofol (Yu et al., 2025a; Chen et al., 2025). However, the potential risks and adverse reactions to ciprofol have not been fully explored, and there is still a lack of direct comparisons among propofol, etomidate, and ciprofol for sedation in the elderly population.

Therefore, we designed a single-center, prospective, double-blind, randomized controlled study to explore the benefits and adverse reactions of propofol, etomidate, and ciprofol for gastrointestinal endoscopy sedation in elderly patients, which might provide a reference for developing sedation plans for elderly patients.

## Methods

This single-center, prospective, double-blind, randomized clinical trial was conducted at Weihai Municipal Hospital following the guidelines for the applicable Consolidated Standards of Reporting Trials (CONSORT) (Schulz et al., 2010) for conducting and reporting clinical trials and the Declaration of Helsinki (World Medical Association, 2013). Ethical approval for this study (approval no.2025035) was approved by the Ethics Committee of Weihai Municipal Hospital on 3 April, 2025. The trial was registered before patient enrollment in the Chinese Clinical Trial Registry (registry no. ChiCTR2500101280, URL: https://www.chictr.org.cn/showproj.html?proj=269837, last updated: 23 April, 2025). Data from elderly patients undergoing gastrointestinal endoscopy between 30 April and 30 December, 2025, were prospectively collected.

### Patients enrollment and visits

The researchers selected elderly patients who met the inclusion criteria, completed the pre-anesthesia assessment, and planned to undergo gastrointestinal endoscopy. Written informed consent was obtained from all patients prior to enrollment. In accordance with the requirements of the research institution, written informed consent was also obtained from the patients’ guardians. Patients and their guardians will be fully aware of the potential benefits and risks of participating in the study and will be informed that they can request withdrawal from the study at any stage without affecting their clinical treatment. Written consent was obtained in a private setting according to the institutional guidelines.

The inclusion criteria were as follows: elderly patients undergoing painless gastrointestinal endoscopy, age ≥ 60 years, ASA I-III, and no serious complications, such as unstable angina pectoris and severe hypertension. Exclusion criteria were as follows: history of major surgery such as cardiac surgery, craniotomy, or abdominal surgery; contraindications or history of allergy to any drugs used in the study; patients with mental illness or cognitive impairment who could not cooperate with follow-up; and long-term use of opioid analgesics or psychotropic drugs. Patient participation will be suspended if they withdraw their informed consent form, major perioperative adverse events occur, do not follow the established anesthesia plan for anesthesia, change the surgical method, shorten or extend endoscopy duration (>60 min or <5 min), or perform other surgeries simultaneously.

### Sample size estimation and random grouping

The patients were randomized in a 1:1:1 ratio into three groups using a block randomization method: propofol (Guangdong Jiabo Pharmaceutical Co., Ltd., Guangdong Province, China) anesthesia group (group P), etomidate (Jiangsu Nhwa Pharmaceutical Co., Ltd., Jiangsu Province, China) anesthesia group (group E), and ciprofol (Haisco Pharmaceutical Group Co., Ltd., Liaoning Province, China) anesthesia group (group C). Computer-generated random sequences was used. We set the block length to alternate between six, and the patients entered the blocks in sequence based on their enrollment time. Within each block, six random numbers were generated sequentially based on the length of the block. Two participants with smaller random numbers were divided into group P, two participants with larger random numbers were included in group C, and the remaining participants were divided into group E. Randomized grouping was performed by a dedicated researcher and sealed in an opaque envelope until the patient entered the operating room. This was a double-blind study, and participants were unaware of the grouping. After the patient entered the operating room, a researcher designated by the research group opened the envelope, prepared the corresponding sedatives, and performed anesthesia induction. During anesthesia induction process, other researchers (including endoscopist, PACU nursing staff, outcome assessors, and all other relevant research personnel) cannot directly observe the anesthesia inducing drugs, avoiding inferring subject grouping and reducing subjective assessment bias. Subsequently, this researcher was no longer responsible for the perioperative management nor involved in data collection and statistical analysis to ensure the implementation of blinding.

It was reported that in gastrointestinal endoscopy anesthesia, the incidence of apnea after propofol anesthesia was more than 40%, while it was 14% for etomidate and 1.4% for ciprofol (Meng et al., 2016; Li et al., 2022). We estimated the incidence of apnea as 30% in group P, 15% in group E, and 5% in group C. Using PASS software, α was set as 0.01, 1- β was set as 0.95, and 81 patients in each group were enrolled. Considering a 10% loss rate, at least 270 patients were included in the study.

### Perioperative management

After entering the operating room, oxygen inhalation was performed (end-tidal carbon dioxide monitoring nasal catheter oxygen inhalation, oxygen inhalation flow 3 L/min) and no preoperative medication was administered. During anesthesia induction, all patients were given sufentanil 0.1 ug/kg. Subsequently, 1.5–2.5 mg/kg of propofol, 0.3–0.6 mg/kg of etomidate and 0.3–0.5 mg/kg of ciprofol were admitted according to the grouping. The injection time was >1 min, and the patients were evaluated until an MOAA/s score ≤1 or the maximum dosage was reached.

Intraoperative vital signs were monitored, and the depth of anesthesia was maintained by intravenous infusion of propofol (4–12 mg/kg/h). If the MOAA/s score was more than 1 or the endoscope insertion failed for two times, propofol 0.3–0.5 mg/kg was injected as rescue sedation. In case of cough, body movement, speech and other reactions during the examination, propofol 0.3–0.5 mg/kg will be added. If the patient had hypotension (systolic blood pressure<90 mmHg or<80% of the baseline value at any time point), dopamine (2 mg) was administered intravenously, and the dose of propofol was reduced by 1 mg/kg/h; if the patient had hypoxemia (SpO_2_ < 96%) during the operation, the mandible was supported first; if the patient had respiratory depression (SpO_2_ < 90%) or apnea, artificial assisted ventilation was used; if the patient had bradycardia (heart rate<50 beats per minute), atropine (0.5 mg) was administered intravenously, and the dose of propofol was reduced by 1 mg/kg/h.

Intravenous infusion of propofol was stopped after the operation and patients were sent to the post-anesthesia care unit (PACU) for continuous observation. All patients were monitored until the modified Aldrete score was ≥9, after which they were allowed to leave the hospital (Aldrete, 1995).

### Outcomes measurement

All data were collected by the designated researcher of the research group who would not participate in randomization, blinding, study drug configuration, or data statistical analysis. The primary outcome was intraoperative respiratory depression, defined as hypoxemia (SpO_2_<96%) and/or apnea (End-tidal carbon dioxide (ETCO_2_) waveform disappears for >5s) at any time during anesthesia. Secondary outcomes included systolic blood pressure (SBP), diastolic blood pressure (DBP), mean blood pressure (MBP), heart rate (HR), respiratory rate (RR), and SpO_2_ at five time points: entry into the operating room (T1), completion of anesthesia induction (T2), after the start of surgery (endoscopy insertion) (T3), 5 min after the start of surgery (T4), and end of surgery (T5); anesthetic-induced injection pain (Numeric Rating Scales, NRS; 0-10)) and myoclonus; bucking, body movement, hiccup, and speech during examination; intraoperative bradycardia and/or hypotension; anesthesia recovery time and Richmond agitation and sedation scale (RASS) score after recovery (Sessler et al., 2002); postoperative nausea and vomiting (PONV) (Gan et al., 2020); dizziness after anesthesia recovery (defined as new-onset heavy-headedness, vertigo or unsteadiness after full orientation recovery in the PACU, evaluated by standardized questioning, and recorded as binary variable); and agitation in the PACU.

### Statistical analysis

Statistical analyses were conducted by a designated researcher who was not involved in any part of the study implementation process. Data analysis followed the Per-Protocol Set (PPs), and missing data were handled using pairwise deletion. For primary outcome analysis, modified intention-to-treat (mITT) was used for robustness analysis, defined as patients who receive predetermined anesthesia induction protocol after grouping, while missing variables were handled by cold deck imputation with nearest-neighbor matching. Normally distributed continuous variables were expressed as mean ± standard deviation and compared using one-way analysis of variance (ANOVA). Non-normally distributed continuous variables were presented as median (interquartile range) and analyzed with the Kruskal-Wallis H test. Categorical data are presented as numbers and were analyzed using the χ2 or Fisher’s exact test, as appropriate. Linear mixed-effects models (LMM) were used to analyze hemodynamic and respiratory monitoring parameters. Group E was set as the reference group for evaluating main group effects, and time point T5 served as the reference for time-by-group interaction effects. Baseline hemodynamic and respiratory parameters, ASA classification, BMI, Mallampati grade, age, and propofol consumption were incorporated as covariates in the models. For effect size estimation, absolute risk difference with 95% CI was used for categorical variables. Among continuous variables, median difference with 95% CI was applied to non-normal data, while mean difference with 95% CI was adopted for normally distributed data. Statistical significance was set at p < 0.05. For multiple comparisons, Bonferroni correction was used to adjust the significance level. The results were analyzed using IBM SPSS Statistics for Windows (version 27; IBM Corporation, USA).

## Results

**FIGURE 1:**
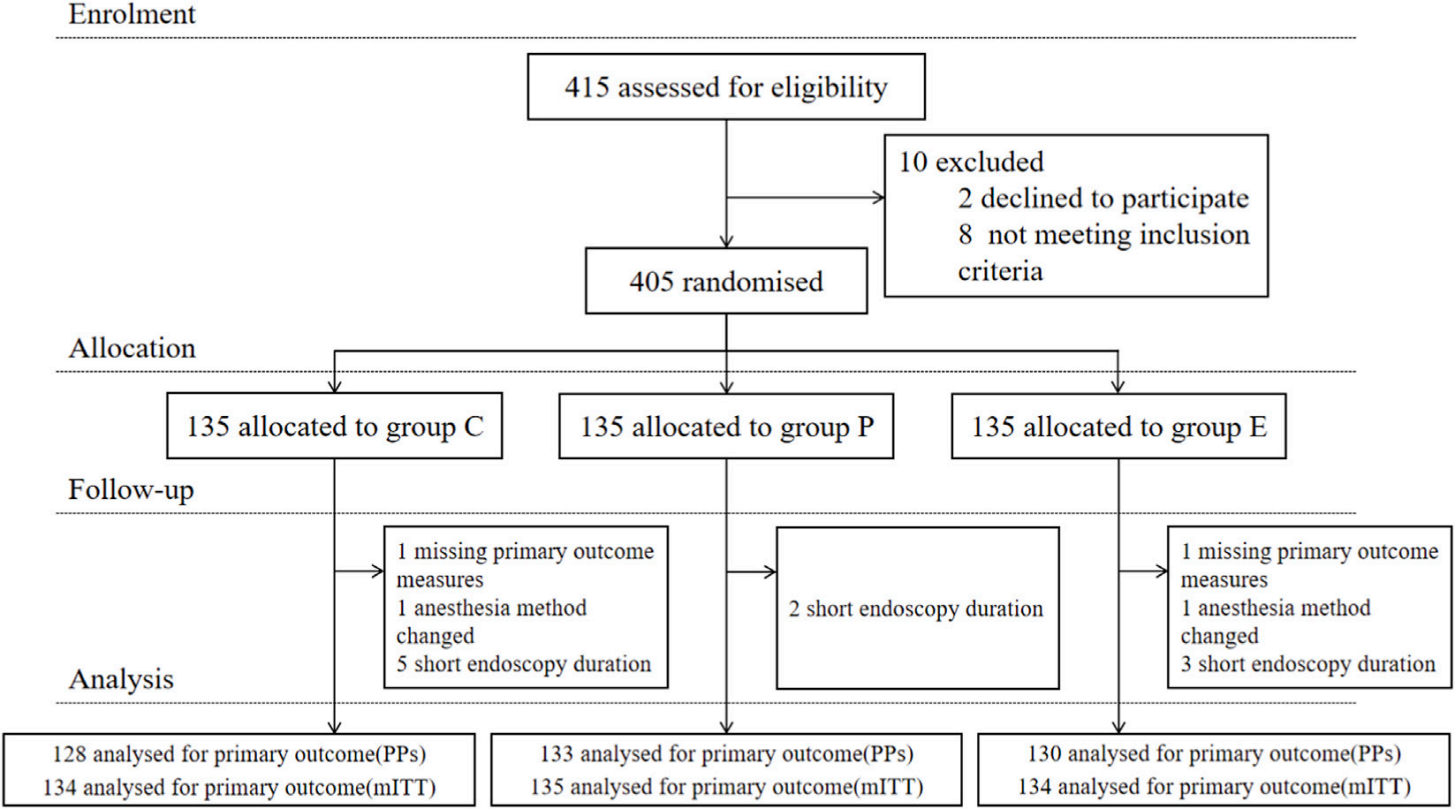
CONSORT flow diagram. Notes: Group C: Ciprofol anesthesia group; Group P: Propofol anesthesia group; Group E: Etomidate anesthesia group.

A total of 415 patients were included in this study. Two patients declined to participate and eight patients did not meet the inclusion criteria, and 405 patients were randomized, with 135 patients in each group. In group C, the primary outcome measures were missed in one patient, endoscopy duration was short in five patients (less than 5 min), and the anesthesia method was changed to local anesthesia in one patient; in group P, endoscopy duration was short in two patients; in group E, primary outcome measures were missed in one patient, endoscopy duration was short in three patients, and the anesthesia method was changed to local anesthesia in one patient. Finally, 391 patients were analyzed, with 128 in group C, 133 in group P, and 130 in group E (Figure 1). The demographic characteristics of the patients, including age, sex, body mass index (BMI), American Society of Anesthesiologists (ASA) physical status classification, Mallampati classification, surgical history, propofol requirement for anesthetic maintenance and rescue sedation, and laboratory and electrocardiogram (ECG) findings, were not significantly different between the groups (Table 1).

**TABLE 1:** Characteristics of participants before surgery.

Variables	Group C (n = 128)	Group P (n = 133)	Group E (n = 130)	p
Age (years)	66.5 (8.0)	67.0 (9.0)	65.0 (8.0)	0.679
Gender (n (male/female))	65/63	65/68	78/52	0.158
ASA (n (%))	​	​	​	0.528
I	10 (7.8)	16 (12.0)	10 (7.7)	​
II	113 (88.3)	113 (85.0)	118 (90.8)	​
III	5 (3.9)	4 (3.0)	2 (1.5)	​
Mallampati grade (n (%))	​	​	​	0.079
I	45 (35.2)	52 (39.10)	38 (29.2)	​
II	78 (60.93)	81 (60.9)	88 (67.7)	​
III	5 (3.9)	0 (0.0)	4 (3.1)	​
BMI (kg/m^2^)	24.43 (4.00)	24.46 (4.53)	25.26 (5.32)	0.456
Comorbidities (n (%))	​	​	​	​
Hypertension	54 (42.2)	58 (43.6)	48 (36.9)	0.511
Diabetes	6 (4.7)	14 (10.5)	6 (4.6)	0.087
Cerebral infarction	2 (1.6)	1 (0.8)	1 (0.8)	0.699
Coronary heart disease	7 (5.5)	9 (6.8)	2 (1.5)	0.088
Endoscopic examination contents (n (%))	​	​	​	0.642
Gastroscope	25 (19.5)	22 (16.5)	23 (17.7)	​
Colonoscope	8 (6.3)	14 (10.5)	9 (6.9)	​
Gastroscope+colonoscope	95 (74.2)	97 (72.9)	98 (75.4)	​
Operation time (minutes)	23.00 (13.75)	25.00 (16.00)	25.00 (18.00)	0.595
Anesthesia duration (minutes)	26.00 (14.75)	29.00 (17.00)	29.00 (18.00)	0.279
Loading induction dose (mg/kg)	0.34 ± 0.08	1.76 ± 0.27	0.38 ± 0.07	—
Propofol consumption during anesthesia maintenance (mg/kg/min)	0.031 ± 0.012	0.032 ± 0.012	0.032 ± 0.011	0.272
rescue propofol requirement (n (%))	4 (3.1%)	2 (1.5%)	6 (4.6%)	0.306
Preoperative laboratory examination	​	​	​	​
RBC (10^12^/L)	4.49 (0.87)	4.57 (1.04)	4.41 (0.54)	0.187
WBC (10^9^/L)	5.65 (2.90)	5.31 (2.47)	5.08 (2.84)	0.615
PLT (10^9^/L)	204.00 (76.75)	214.00 (65.50)	198.00 (83.50)	0.705
HGB (g/L)	140.00 (27.00)	137.00 (27.75)	138.00 (16.50)	0.778
PT (seconds)	11.70 (1.00)	11.50 (1.00)	11.70 (1.00)	0.284
APTT (seconds)	27.71 ± 2.68	27.77 ± 2.24	27.98 ± 2.68	0.969
K^+^ (mmol/L)	4.09 (0.54)	4.07 (0.44)	4.19 (0.55)	0.832
Ca^2+^ (mmol/L)	2.24 (0.17)	2.30 (0.18)	2.23 (0.15)	0.247
Na^+^ (mmol/L)	142.25 (3.25)	142.30 (3.55)	141.90 (4.05)	0.562
Preoperative ECG examination (n (%))	​	​	​	0.633
Normal	70 (68.6)	73 (64.0)	78 (69.6)	​
Abnormal^a^	32 (31.4)	41 (36.0)	34 (30.4)	​

^a^ Abnormal electrocardiograms include sinus tachycardia, sinus bradycardia, sinus arrhythmia, atrial premature beats, and ventricular premature beats.

Abbreviations: ASA: american society of anesthesiologists physical status classification system; BMI:body mass index; RBC: red blood cell count; WBC: white blood cell count; HGB: hemoglobin; PLT: platelet; PT: prothrombin time; APTT: activated partial thromboplastin time.

**FIGURE 2:**
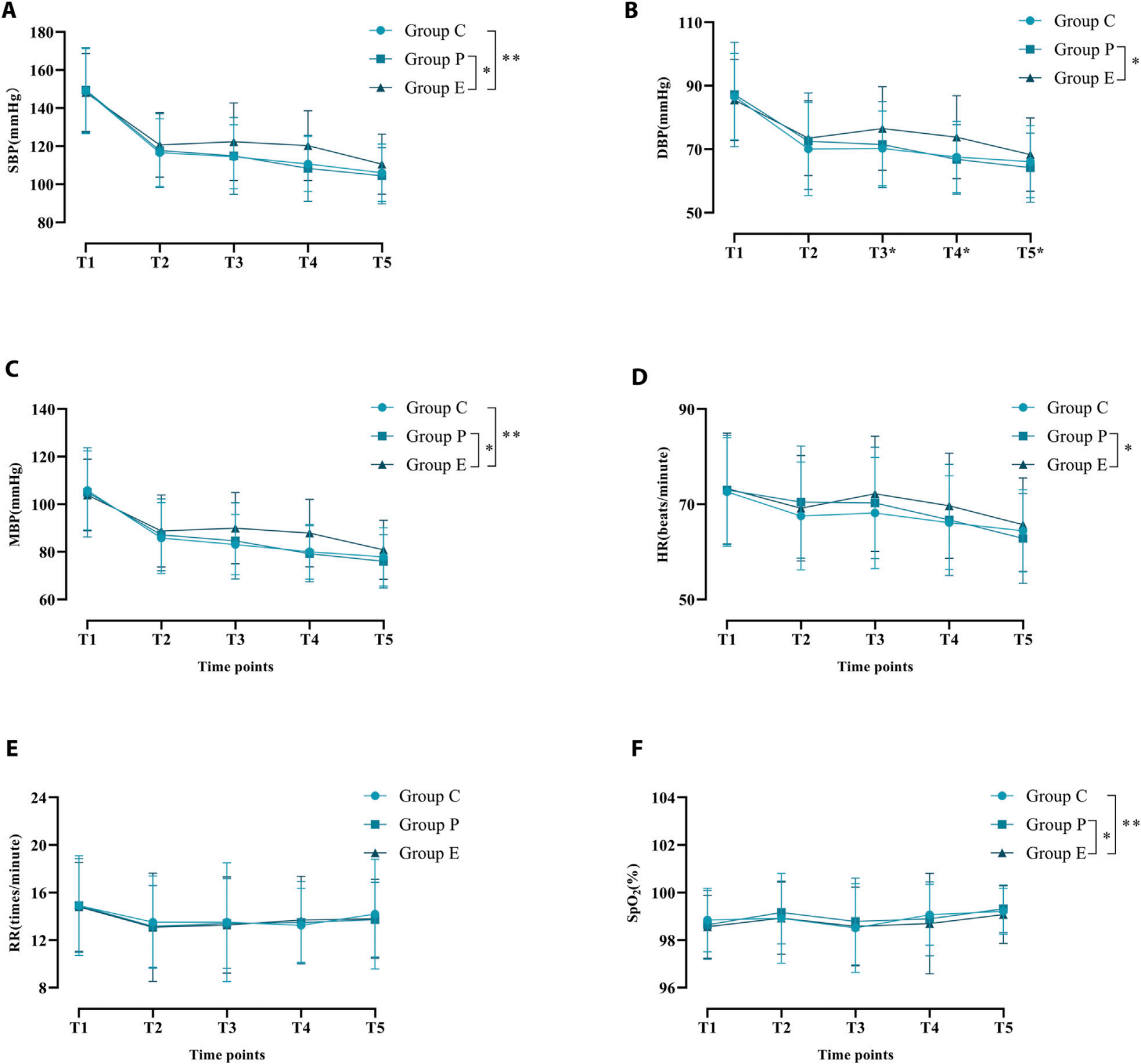
Vital signs at different time point. **(A)** Systolic blood pressure at different time point. **(B)** Diastolic blood pressure at different time point. **(C)** Mean blood pressure at different time point. **(D)** Heart rate at different time point. **(E)** Respiratory rate at different time point. **(F)** SpO2 at different time point. Notes: T1, entry into the operating room; T2, completion of anesthesia induction; T3, after the start of surgery (endoscopy insertion); T4, 5 min after the start of surgery; T5, end of surgery (T5).

While there were slight between-group differences in SpO_2_ values (Figure 2; Supplementary Table S2), no statistically significant differences were detected in the incidences of hypoxemia and apnea across groups (Table 2; Supplementary Table S3). In terms of main effects, both group C and gGroup P exhibited significantly lower SBP and MBP than group E; group P also showed reduced DBP and HR, whereas DBP and HR were not statistically different between group C and group E. (Figure 2; Supplementary Tables S1, S2). The incidence of bradycardia was lower in group P (10.2% vs. 1.5% vs. 5.4%, p = 0.010), whereas the incidence of hypotension was lower in group E (15.6% vs. 20.3% vs. 6.9%, p = 0.007) (Table 2). The proportion of patients requiring vasoactive drugs during surgery was lower in group E (19.5% vs. 21.8% vs. 8.5%, p = 0.006) (Table 2).

**TABLE 2:** Research outcomes.

Variables	Group C (n = 128)	Group P (n = 133)	Group E (n = 130)	P^a^
NRS of injection pain	​	​	​	<0.001*
0	126 (98.4)^a^	56 (42.1)^b^	123 (94.6)^a^	​
1	2 (1.6)^a^	62 (46.6)^b^	6 (4.6)^a^	​
2	0 (0.0)^a^	15 (11.3)^b^	1 (0.8)^a^	​
Myoclonus after induction	6 (4.7)^a^	6 (4.5)^a^	26 (20.0)^b^	<0.001*
Bucking during insertion^b^	12 (10.0)	18 (15.1)	16 (13.2)	0.501
Swallowing during insertion^b^	13 (10.8)	10 (8.4)	16 (13.2)	0.482
Adverse reactions during gastrointestinal endoscopy (n (%))	​	​	​	​
Body movement	12 (9.4)	14 (10.5)	8 (6.2)	0.429
Bucking	9 (7.0)^a^	14 (10.5)^ab^	24 (18.5)^b^	0.015*
Hiccup	3 (2.3)^a^	12 (9.0)^b^	15 (11.5)^b^	0.016*
Speech	1 (0.8)	1 (0.8)	5 (3.8)	0.114
Intraoperative respiratory and hemodynamics (n (%))	​	​	​	​
Bradycardi^a^	13 (10.2)^a^	2 (1.5)^b^	7 (5.4)^ab^	0.010*
Hypotension	20 (15.6)^a^	27 (20.3)^a^	9 (6.9)^b^	0.007*
Hypoxemi^a^	9 (7.1)	10 (7.5)	11 (8.5)	0.914
Apne^a^	5 (3.9)	7 (5.3)	4 (3.1)	0.666
Application of vasoactive drugs	25 (19.5)^a^	29 (21.8)^a^	11 (8.5)^b^	0.006*
Postoperative observation indexes	​	​	​	​
Nausea (n (%))	1 (0.8)	4 (3.0)	1 (0.8)	0.262
Vomiting (n (%))	0 (0)	0 (0)	0 (0)	—
Dizziness (n (%))	6 (4.7)^a^	31 (23.3)^b^	19 (14.6)^b^	<0.001*
Agitation (n (%))	0 (0)	0 (0)	0 (0)	—
Anesthesia recovery time (minutes)	8.5 (6.0)^a^	11.0 (7.0)^b^	8.5 (6.0)^a^	<0.001*
RASS after anesthesia recovery (n (%))	​	​	​	0.031*
−2	0 (0)^a^	0 (0)^a^	1 (0.8)^a^	​
−1	29 (22.8)^a^	17 (12.8)^ab^	15 (11.5)^b^	​
0	98 (77.2)^a^	116 (87.2)^a^	114 (87.7)^a^	​

Abbreviations: NRS, numerical rating scale; RASS, richmond agitation and sedation scale.

^a^ All reported p values are corrected by the Bonferroni method, calculated as the raw p value multiplied by the total number of pairwise comparisons.

^b^ When calculating the percentage and chi-square analysis of outcomes indicators during gastroscopy insertion process, the total number of patients was counted after removing those who only underwent colonoscopy.

An asterisk (*) denotes statistical significance at p < 0.05.

Differences in the incidence of injection pain were also observed. More patients in group P reported NRS scores of 1 and 2 (46.6% and 11.3%, respectively), while more patients in group C and E reported NRS scores of 0 (98.4% and 94.6%, respectively) (p < 0.001) (Table 2). The incidence of myoclonus after anesthesia induction in group E was significantly higher (4.7% vs. 4.5% vs. 20%, p < 0.001), but there was no difference in the incidence of bucking and swallowing during endoscopy. There was no significant difference in intraoperative body movement and speech; however, the incidence of bucking and hiccups in group E was higher (7.0% vs. 10.5% vs. 18.5%, p = 0.015; 2.3% vs. 9.0% vs. 11.5%, p = 0.016) (Table 2). In terms of anesthesia recovery, the RASS scores of all patients were between -2-0 (Table 2). The incidence of dizziness was lower in group C (4.7% vs. 23.3% vs. 14.6%, p < 0.001), but there was no difference in the incidence of PONV and agitation (Table 2).

## Discussion

Maintaining hemodynamic stability is the core of anesthesia management in elderly patients. Studies have shown that hypotension for >5 min during anesthesia in elderly patients is associated with an increased risk of postoperative delirium risk (Duan et al., 2023). Another meta-analysis showed that intraoperative hypotension was associated with postoperative acute kidney injury in elderly patients (Hou et al., 2025). Our study found that patients in the three groups experienced varying degrees of blood pressure and HR decline after anesthesia. The decrease in blood pressure in groups P and C was similar, whereas the decrease in group E was slight. The difference was found after endoscopy insertion and 5 min after the examination, but there was no significant difference in blood pressure and HR at the end of the examination. Considering that all patients received an intravenous infusion of propofol during anesthesia maintenance, and the average values of blood pressure and HR were within the normal range, we believe that with the metabolism of induction anesthetics and the patient’s compensatory reaction, this hemodynamic fluctuation is still controllable. Nevertheless, we also found that although statistically significant intergroup differences were detected in several hemodynamic parameters, the mean differences were mostly around 5 mmHg. Such minor fluctuations are generally clinically acceptable and rarely require targeted intervention. Similarly, intergroup differences in SpO_2_ were approximately 1%. Overall, a difference of this magnitude cannot be considered clinically meaningful and is unlikely to confer improved clinical prognoses for patients.

However, the percentage of vasoactive drugs used in groups P and C was approximately 20%, which was significantly higher than in group E. The incidence of bradycardia was higher in Group C, the incidence of hypotension was higher in Group P, and hypotension and bradycardia were relatively low in Group E. We can see from this that the influence of propofol and ciprofol on hemodynamics in the elderly population may be similar, which is slightly different from the results of other research groups. According to meta-analyses involving the elderly, the incidence of hypotension and bradycardia during gastrointestinal endoscopy anesthesia with ciprofol was lower than that with propofol (Cheng et al., 2025; Liu et al., 2024). Another randomized controlled trial showed that in elderly patients with hip fracture, the fluctuation of blood pressure in the ciprofol induction group was smaller (Lu et al., 2024). We believe that this might be due to the failure to analyze the elderly population independently in the meta-analysis, and that the drugs used in general anesthesia induction are more complex than sedation during gastrointestinal endoscopy. In addition, injection speed may also affect adverse cardiovascular reactions. Our study showed that in the elderly population, ciprofol anesthesia might not be significantly better than propofol in terms of hemodynamic stability and the risk of vasoactive drug application. Clinicians need to closely monitor hemodynamic fluctuations and prepare vasoactive drugs at any time when administering ciprofol to elderly patients. For patients at high risk of hemodynamic fluctuations, etomidate might be more appropriate because its blood pressure decrease after anesthesia induction is smaller and the risk of vasoactive drug application is lower.

Our results showed that there was no significant difference in the incidence of hypoxemia and apnea among the three groups, which is different from the results of other studies. A randomized controlled study by Wang et al. (2024) showed that the incidence of hypoxemia under propofol anesthesia decreased from 9.8% to 3.3% in endoscopic retrograde cholangiopancreatography (ERCP). Another meta-analysis also showed that compared with propofol, ciprofol significantly reduced the incidence of respiratory depression during gastrointestinal endoscopy anesthesia (OR = 0.21, 95% CI: 0.15–0.30) (Cheng et al., 2025). The reason that our study did not reach a positive result might be that we specifically included the elderly group. The factors affecting respiration are complex, and it is difficult to produce clinically significant changes in narcotic drugs. Elderly individuals have decreased vital capacity and expiratory flow, increased residual volume and ventilation perfusion heterogeneity, weakened respiratory muscle strength, reduced lung reserve, and increased sensitivity to respiratory depression (Sprung et al., 2006), which might lead to various pulmonary complications in the elderly during the perioperative period. Simultaneously, we performed a slower drug injection speed during anesthesia induction, and all patients received a unified and low propofol pump speed; therefore, the incidence of apnea and hypoxemia in all groups was less than 10%, which was similar to the results of other studies. However, we did not choose to pump etomidate or ciprofol during the anesthesia maintenance phase because of the obvious price advantage of propofol in China. We hope that our research results can be popularized in follow-up clinical practice without increasing medical burden. Therefore, we cannot determine whether the use of ciprofol or etomidate in anesthesia maintenance can reduce the incidence of hypoxemia and apnea, which is a limitation of our study. Further clinical research is needed to address this issue.

In terms of satisfaction with anesthesia induction, ciprofol has obvious advantages. Our study found that both ciprofol and etomidate had a lower incidence of injection pain; however, incidence of myoclonus was lower after ciprofol induction than etomidate. This suggests that ciprofol can induce satisfactory anesthesia and reduce the incidence of adverse reactions. At the same time, the incidence of bucking and hiccups was lowest in group C, which suggests that ciprofol could provide ideal sedation levels and improve the satisfaction of clinicians. In terms of anesthesia recovery, the incidence of dizziness was the lowest in group C. However, not all studies have supported this result. A meta-analysis indicated that the proportion of postoperative dizziness was approximately 30%, regardless of propofol or ciprofol sedation (Yu et al., 2025b). Age, sex, educational level, endoscopic surgery, and long-acting opioids are risk factors for postoperative dizziness (Chin et al., 2025; Zhou et al., 2025). Considering the significant effect of ciprofol in reducing dizziness in our study, we believe that it might be the preferred drug for elderly patients with postoperative dizziness risk; however, further research is still needed to explore its effectiveness and applicable scenarios. In addition, the incidence of PONV and other adverse reactions among the groups was similar; the RASS score after recovery was mostly −1 and 0, and no serious adverse events such as cardiovascular and cerebrovascular accidents occurred during the perioperative period, suggesting that the three anesthetic drugs can be safely and effectively used in the anesthesia management of gastrointestinal endoscopy in elderly patients.

Clinical interpretation of effect sizes was performed (see Supplementary Table S4). For adverse events, group C and group P exhibited a approximately 15% reduction in the risk of myoclonus after induction relative to group E. The incidence of postoperative dizziness was 18.6% lower in group C vs. group P, representing a prominent clinical advantage for patient comfort. However, group P carried a 13.4% higher risk of intraoperative hypotension compared with group E, which merits attention to hemodynamic stability during endoscopy. In terms of continuous endpoints, the median difference of 1 point in NRS injection pain score in group P was a perceptible clinical distinction, and group P had a 2.5-min longer anesthesia recovery time. Collectively, the three induction regimens demonstrated clinically distinguishable performance in induction-related adverse events, injection pain and recovery duration, while comparable effects were observed in hemodynamic intervention requirements.

Our study has some limitations. First, we did not conduct a subgroup analysis, such as sex, age, and type of examination, so we could not obtain the best application population and type of examination for the three sedatives. Second, we did not collect blood samples from patients; therefore, we could not reveal the mechanism of the above differences. Third, considering that narcotic drugs cause anterograde amnesia, the result of the injection pain might deviate from the actual situation. Exploring a more accurate evaluation method for injection pain is helpful for reducing bias and reflecting the real situation more objectively. Fourth, we included fewer older adults, and most patients were between 60 and 75 years old. Therefore, which of the three drugs is more suitable for anesthesia management in elderly people still needs further study. Fifth, propofol was used uniformly for anesthetic maintenance and rescue sedation across all three groups. Although no statistically significant differences were observed in the dosage of maintenance anesthetics and the demand for rescue sedation, this design may attenuate the interpretability of discrepancies in outcome indicators attributable to distinct anesthesia induction regimens, such as respiratory depression, anesthesia recovery time, and post-anesthesia dizziness. Sixth, due to insufficient preliminary research data, the sample size calculation in this study only adopted apnea as a single indicator without incorporating the index of low SpO_2_, which fails to meet the requirements for sample size estimation based on composite outcome endpoints. In addition, the incidences of reported outcome indicators were lower than the prespecified values used for sample size calculation, hence the negative results may stem from inadequate statistical power. For subsequent studies, sample size calculation could be performed based on this research. Multi-center, large-sample clinical trials might be recommended with enrollment of more advanced-age elderly patients and adoption of a consistent medication strategy. Biological specimens might be collected for laboratory analysis when appropriate, so as to further explore the clinical characteristics of the three anesthetic agents used in gastrointestinal endoscopy anesthesia among elderly patients and provide higher-level clinical evidence for this field.

## Conclusion

When used as anesthesia induction drugs, propofol, etomidate, and ciprofol are optional sedatives for elderly patients undergoing gastrointestinal endoscopy. Etomidate has the advantages of stable hemodynamics and low incidence of injection pain; however, the incidence of myoclonus after induction, bucking, and hiccups during the examination is high. The incidence of injection pain, bucking, and hiccups during the examination and postoperative dizziness was lower under ciprofol anesthesia. Although the anesthetic effect of propofol is ideal, the incidence of injection pain is high. No significant differences were observed in the incidence of perioperative hypoxemia and apnea, and the time and quality of anesthesia recovery were similar. Clinicians can choose the appropriate drugs according to the patient’s situation, formulate personalized anesthesia programs, and improve patient benefits.

## Data Availability

The raw data supporting the conclusions of this article will be made available by the authors, without undue reservation.
