# Supplementary material for: Comparison of anesthesia induction with ciprofol, propofol, and etomidate in elderly patients undergoing gastrointestinal endoscopy: a single center, prospective, double blind randomized controlled trial
Source: Front Aging. 2026 Aug 3;7:1842800. doi: 10.3389/fragi.2026.1842800 (PMC13478072; doi:10.3389/fragi.2026.1842800)
Supplement: Supplementary file 1 [file Table1.docx]

**Table S1 Hemodynamic and Respiratory Monitoring Parameters**

|  | Group C  (n=128) | Group P  (n=133) | Group E  (n=130) |
| --- | --- | --- | --- |
|  |  |  |  |
| SBP(mmHg) | | | |
| T0 | 148.93±22.32 | 149.45±22.37 | 148.23±20.50 |
| T1 | 116.66±17.81 | 117.73±19.38 | 120.66±17.02 |
| T2 | 114.46±16.81 | 114.98±20.23 | 122.37±20.39 |
| T3 | 110.67±14.47 | 108.42±17.39 | 120.36±18.33 |
| T4 | 106.05±15.08 | 104.52±14.75 | 110.61±15.72 |
| DBP(mmHg) | | | |
| T0 | 86.57±13.61 | 87.26±16.45 | 85.50±12.81 |
| T1 | 70.08±14.67 | 72.55±15.26 | 73.48±11.79 |
| T2 | 70.27±11.79 | 71.48±13.55 | 76.51±13.14 |
| T3 | 67.52±11.23 | 66.78±10.96 | 73.82±13.05 |
| T4 | 66.08±11.31 | 64.18±10.89 | 68.28±11.56 |
| MBP(mmHg) | | | |
| T0 | 105.65±16.64 | 105.03±18.77 | 103.91±15.06 |
| T1 | 85.80±14.90 | 87.11±15.16 | 88.80±15.17 |
| T2 | 83.13±12.74 | 84.61±16.00 | 89.98±15.00 |
| T3 | 80.06±11.51 | 79.21±11.81 | 87.91±14.17 |
| T4 | 77.87±12.33 | 76.03±11.21 | 80.87±12.36 |
| HR(beats per minute) | | | |
| T0 | 72.62±11.40 | 73.00±11.45 | 73.31±11.64 |
| T1 | 67.54±11.33 | 70.45±11.33 | 69.17±11.07 |
| T2 | 68.17±11.69 | 70.28±11.72 | 72.21±12.12 |
| T3 | 66.13±9.85 | 66.72±11.68 | 69.68±11.03 |
| T4 | 64.44±8.63 | 62.82±9.45 | 65.72±9.81 |
| RR(times per minute) | | | |
| T0 | 14.91±4.19 | 14.91±3.95 | 14.80±3.74 |
| T1 | 13.52±3.88 | 13.16±3.44 | 13.08±4.55 |
| T2 | 13.52±4.99 | 13.41±3.79 | 13.27±4.05 |
| T3 | 13.25±3.11 | 13.48±3.46 | 13.70±3.68 |
| T4 | 14.20±4.61 | 13.73±3.14 | 13.80±3.33 |
| SpO_2_(%) | | | |
| T0 | 98.85±1.33 | 98.65±1.45 | 98.56±1.32 |
| T1 | 98.92±1.89 | 99.17±1.32 | 98.93±1.52 |
| T2 | 98.51±1.87 | 98.79±1.82 | 98.58±1.65 |
| T3 | 99.07±1.28 | 98.90±1.56 | 98.70±2.11 |
| T4 | 99.22±0.97 | 99.33±1.00 | 99.08±1.22 |
