# Supplementary material for: Comparison of anesthesia induction with ciprofol, propofol, and etomidate in elderly patients undergoing gastrointestinal endoscopy: a single center, prospective, double blind randomized controlled trial
Source: Front Aging. 2026 Aug 3;7:1842800. doi: 10.3389/fragi.2026.1842800 (PMC13478072; doi:10.3389/fragi.2026.1842800)
Supplement: Supplementary file 2 [file Table2.docx]

**Table S2 Results of Linear Mixed-effects Models Analysis for Hemodynamic and Respiratory Monitoring Parameters**

|  | | **SBP(mmHg)** | | **DBP(mmHg)** | | **MBP(mmHg)** | | **HR(times/minutes)** | | **RR(times/minutes)** | | **SpO_2_(%)** | |
| --- | --- | --- | --- | --- | --- | --- | --- | --- | --- | --- | --- | --- | --- |
|  |  | MD(95%CI) | p | MD(95%CI) | p | MD(95%CI) | p | MD(95%CI) | p | MD(95%CI) | p | MD(95%CI) | p |
| **Main Effect (compared to Group E)** | | | | | | | | | | | | | |
| **Group C-Group E** | | -4.61(-8.48, -7.47) | 0.019 | -2.48(-5.25, -0.29) | 0.080 | -3.50(-6.52, -0.48) | 0.023 | -0.68(-2.72, 1.36) | 0.513 | 0.38(-0.55, 1.31) | 0.423 | 0.72(0.15, 1.30) | 0.014 |
| **Group P-Group E** | | -5.97(-9.80, -2.13) | 0.002 | -4.37(-7.12, -1.61) | 0.002 | -4.76(-7.75, -1.76) | 0.002 | -2.32(-4.35, -0.31) | 0.024 | -0.03(-0.95, 0.90) | 0.952 | 0.82(0.25, 1.39) | 0.005 |
| **Interaction Effect (time*group) (compared to Group E and T5)** | | | | | | | | | | | | | |
| **Group C** | T2 | 0.59(-3.51, 4.70) | 0.777 | -1.15(-3.96, 1.65) | 0.419 | -0.04(-2.95, 3.03) | 0.980 | -0.34(-2.28, -1.61) | 0.734 | 0.03(-1.12, -1.17) | 0.962 | -0.85(-1.59, -0.11) | 0.024 |
|  | T3 | -3.29(-7.39, 0.82) | 0.117 | -4.02(-6.82, -1.22) | 0.005 | -3.87(-6.86, -0.88) | 0.011 | -2.74(-4.79, -0.89) | 0.004 | -0.19(-1.33, 0.95) | 0.748 | -0.92(-1.66, -0.18) | 0.015 |
|  | T4 | -5.26(-9.36, -1.15) | 0.012 | -4.16(-6.96, -1.36) | 0.004 | -4.93(-7.91, -1.95) | 0.001 | -2.34(-4.29, -0.40) | 0.018 | -0.88(-2.02, 0.27) | 0.133 | -0.46(-1.21, 0.29) | 0.219 |
| **Group P** | T2 | 3.20(-0.86, 7.27) | 0.123 | 3.24(0.47, 6.02) | 0.022 | 3.22(0.26, 6.17) | 0.033 | 4.17(2.25, 6.11) | <0.001 | 0.14(-0.99, 1.28) | 0.803 | -0.72(-1.45, 0.02) | 0.058 |
|  | T3 | -1.15(-5.22, 2.92) | 0.579 | -0.88(-3.66, 1.89) | 0.532 | -0.48(-3.43, -2.47) | 0.749 | 0.80(-1.13, -2.73) | 0.417 | 0.15(-0.98, 1.29) | 0.790 | -0.76(-1.49, -0.02) | 0.043 |
|  | T4 | -6.06(-10.12, -1.99) | 0.004 | -3.06(-5.83, -0.28) | 0.031 | -3.99(-6.95, -1.04) | 0.008 | -0.20(-2.12, -1.73) | 0.842 | -0.81(-1.32, 0.95) | 0.747 | -0.73(-1.48, 0.01) | 0.052 |
