# Supplementary material for: Comparison of anesthesia induction with ciprofol, propofol, and etomidate in elderly patients undergoing gastrointestinal endoscopy: a single center, prospective, double blind randomized controlled trial
Source: Front Aging. 2026 Aug 3;7:1842800. doi: 10.3389/fragi.2026.1842800 (PMC13478072; doi:10.3389/fragi.2026.1842800)
Supplement: Supplementary file 3 [file Table3.docx]

**Table S3 Robustness Analysis of Primary Outcome Indicators**

| **Variables** | **Group C**  **(n=134)** | **Group P**  **(n=135)** | **Group E**  **(n=134)** | **p** |
| --- | --- | --- | --- | --- |
| Hypoxemia | 10(7.5%) | 10(7.4%) | 11(8.2%) | 0.963 |
| Apnea | 6(4.5%) | 7(5.2%) | 4(3.0%) | 0.646 |
