# Supplementary material for: Comparison of anesthesia induction with ciprofol, propofol, and etomidate in elderly patients undergoing gastrointestinal endoscopy: a single center, prospective, double blind randomized controlled trial
Source: Front Aging. 2026 Aug 3;7:1842800. doi: 10.3389/fragi.2026.1842800 (PMC13478072; doi:10.3389/fragi.2026.1842800)
Supplement: Supplementary file 4 [file Table4.docx]

**Table S4 Effect Size of Outcome Indicators**

| **Variables** | **Group C vs. Group E** | **Group P vs. Group E** | **Group C vs. Group P** |
| --- | --- | --- | --- |
| Myoclonus after induction^1^ | -0.153(-0.231,-0.075) | -0.155(-0.232,-0.078) | 0.002(-0.049,0.039) |
| Intraoperative bucking^1^ | -0.115(-0.195,-0.035) | -0.080(-0.165,0.005) | -0.035(-0.103,0.033) |
| Intraoperative hiccup^1^ | -0.115(-0.153,-0.031) | -0.025(-0.098,0.048) | -0.067(-0.122,-0.012) |
| Intraoperative bradycardia^1^ | 0.048(-0.017,0.113) | -0.039(-0.083,0.005) | 0.087(0.031,0.143) |
| Intraoperative hypotension^1^ | 0.087(0.011,0.163) | 0.134(0.053,0.215) | -0.047(-0.140,0.046) |
| Application of vasoactive drugs^1^ | 0.110(0.026,0.194) | 0.133(0.048,0.218) | -0.023(-0.0.121,0.0759) |
| Postoperative dizziness^1^ | -0.099(-0.028,-0.007) | 0.087(-0.007,0.181) | -0.186(-0.267,-0.105) |
| NRS of injection pain^2^ | 0.00(0.00,0.00) | 1.00(0.73,1.27) | -1.00(-1.27,-0.73) |
| Anesthesia recovery time^2^(minutes) | 0.00(-2.30,2.30) | 2.50(0.03,4.97) | -2.5,(-4.98,-0.02) |
| RASS after anesthesia recovery^2^ | 0.00(0.00,0.00) | 0.00(0.00,0.00) | 0.00(0.00,0.00) |

Notes: 1: For categorical variables, the absolute risk difference (ARD) along with its 95% confidence interval (95%CI) was calculated; 2: For continuous variables that failed to conform to a normal distribution, pairwise median differences and corresponding 95% confidence intervals were estimated.
